# Stochastic growth pattern of untreated human glioblastomas predicts the survival time for patients

**DOI:** 10.1038/s41598-020-63394-w

**Published:** 2020-04-20

**Authors:** Ziwei Ma, Ben Niu, Tuan Anh Phan, Anne Line Stensjøen, Chibawanye Ene, Timothy Woodiwiss, Tonghui Wang, Philip K. Maini, Eric C. Holland, Jianjun Paul Tian

**Affiliations:** 1 Department of Mathematical Sciences, New Mexico State University, 1780 E University Ave, Las Cruces, NM 88003 USA; 2 Human Biology Division, Fred Hutchinson Cancer Research Center, 1100 Fairview Avenue N., PO Box 19024, Seattle, WA 98109 USA; 3 Solid Tumor Translational Research, Fred Hutchinson Cancer Research Center, 1100 Fairview Avenue N., PO Box 19024, Seattle, WA 98109 USA; 4 Department of Mathematics, Harbin Institute of Technology at Weihai, 2 West Wenhua Road, Weihai, Shandong 264209 P.R. China; 5 College of Sciences, Northwest A&F University, 22 Xinong Rd, Yangling, Shaanxi 712100 China; 6 Department of Circulation and Medical Imaging, Faculty of Medicine and Health sciences, NTNU - Norwegian University of Science and Technology, Post Box 8905, N-7491 Trondheim, Norway; 7 Wolfson Centre for Mathematical Biology, Mathematical Institute, University of Oxford, Woodstock Road, Oxford, OX2 6GG UK

**Keywords:** Computational models, Cancer models

## Abstract

Glioblastomas are highly malignant brain tumors. Knowledge of growth rates and growth patterns is useful for understanding tumor biology and planning treatment logistics. Based on untreated human glioblastoma data collected in Trondheim, Norway, we first fit the average growth to a Gompertz curve, then find a best fitted white noise term for the growth rate variance. Combining these two fits, we obtain a new type of Gompertz diffusion dynamics, which is a stochastic differential equation (SDE). Newly collected untreated human glioblastoma data in Seattle, US, re-verify our model. Instead of growth curves predicted by deterministic models, our SDE model predicts a band with a center curve as the tumor size average and its width as the tumor size variance over time. Given the glioblastoma size in a patient, our model can predict the patient survival time with a prescribed probability. The survival time is approximately a normal random variable with simple formulas for its mean and variance in terms of tumor sizes. Our model can be applied to studies of tumor treatments. As a demonstration, we numerically investigate different protocols of surgical resection using our model and provide possible theoretical strategies.

## Introduction

Glioblastomas are highly aggressive primary malignant brain tumors. They have a very poor prognosis. For most types of glioblastomas, there has been minimal improvement in survival in the past decades^1,2^. Knowledge of glioma growth rates and underlying growth dynamics is important for understanding basic tumor biology, developing realistic tumor models, and planning treatment logistics. However, the growth dynamics of human untreated glioblastomas *in vivo* has not been studied in detail^3^. The main reason is that we do not have relevant data that can be used to calculate tumor growth rates and to discover tumor growth patterns *in vivo* for humans, although we have a large data pool from experiments with animal models.

Tumor growth is the outcome of the complex interactions among tumor cells and their microenvironment, where cell proliferation, blood and nutrition supply, and cell death (i.e., apoptosis and necrosis) are also influenced by many environmental features^4^. In spite of a large set of potential parameters, some tumors have a particular growth pattern that can be described by a Gompertzian curve^5^, which often is considered as a purely phenomenological growth curve. Namely, there is an initial exponential growth, and then it is followed by a saturation phase^6^. The Gompertz growth model seems to have some predictive power since it characterizes a common growth pattern of fast growth in the beginning and slowdown to a maximum size. The Gompertz model has been applied to several types of solid tumors^7^. However, the Gompertz growth model often exhibits discrepancies between clinical or experimental data and theoretical predictions^8^. These discrepancies may be due to internal environmental fluctuations and variation among patients. To consider such environmental fluctuations and variation in individual patients, some tumor growth models incorporate stochastic processes. For example, Albano & Giorno^9^ and Lo^10^ considered some stochastic models for tumor growth.

In a recent clinical study, Stensjøen *et al*. reported a data set of untreated human glioblastoma *in vivo*^3^. They calculated the tumor growth rates for each patient, and found considerable variation among individual patients.

In this study, we propose a Gompertz diffusion growth model that is the best fit to the data set of 94 untreated glioblastoma patients collected in the aforementioned study conducted by Stensjøen *et al*.^3^ in Trondheim, Norway. Our model is a stochastic differential equation, where white noise captures the variants of glioblastoma growth among patients. Our model is re-verified by newly collected data sets from Seattle, US. By computer simulations, we find that the patient survival time is a normally distributed random variable and its mean and variance are explicit functions of current tumor volumes. Using our model, we can predict the survival time for each patient based on a magnetic resonance imaging (MRI) scan with a prescribed probability before treatments.

As proof of principle, we conduct a theoretical study of surgical resection using our mathematical model. The extent of resection^11–15^ and repeated resections^16,17^ have been important subjects in neurosurgery clinics and research. We will study a variety of resection protocols including extent and repeated resections. In particular, we will study the following questions. (1) How long could a patient survive without treatment given the initial diagnosed tumor size? (2) How long could a patient survive if the patient accepts surgical resection immediately after diagnosis of glioblastoma? (3) How long could a patient survive if the patient accepts surgical resection after some period of time after diagnosis of glioblastoma? (4) If a second surgical resection is performed, how long could a patient survive? Our computational study will answer these questions in detail and provide some suggestions which may help surgeons to determine optimal treatment strategies. Computationally, we confirm three established conclusions: patients with small glioblastomas have a longer survival time while large glioblastomas have a short survival time^11,12^; the patient will have survival benefits even for 50% of the extent of resection^13,14^; repeated resections will increase patient survival time^16,17^. Quantifying the extent of resection, we obtain the following theoretical conclusions. For large glioblastomas, the more a surgical resection can cut off from the tumor, the longer the survival time for the patient. For small glioblastomas, if we wait until the glioblastoma grows to a large tumor and then perform surgical resection, the patient survival time will increase; furthermore, if we wait again until the glioblastoma grows to an even larger tumor and then perform a second surgical resection, the patient survival time will increase further. However, these conclusions are purely theoretical outcomes which do not consider many medical factors, and therefore may not be immediately applicable in the clinics as many other aspects of the tumor need to be taken into account.

## Methods

In the study conducted by Stensjøen *et al*.^3^, 106 untreated human glioblastoma data sets were collected. In that study, there are tumor volumes from two MRI scans for each patient, and the time between the two MRIs. These measurements were taken before surgery. Specifically, for the i-th patient, we have the record $({{\rm{V}}}_{1}^{{\rm{i}}},{\Delta {\rm{t}}}^{{\rm{i}}},{{\rm{V}}}_{2}^{{\rm{i}}})$, where ${{\rm{V}}}_{1}^{{\rm{i}}},\,{\rm{and}}\,{{\rm{V}}}_{2}^{{\rm{i}}}$ represent the tumor size of the first and second MRI scan, respectively, and ${\Delta {\rm{t}}}^{{\rm{i}}}$ represents the time between the two MRI scans, ${\rm{i}}=1,\cdots ,94$. We choose to work on 94 glioblastoma data sets because these tumor sizes increase over the measured time period. The unit of tumor size is mL. The unit of time is day.

### Growth estimations

**Figure 1:**
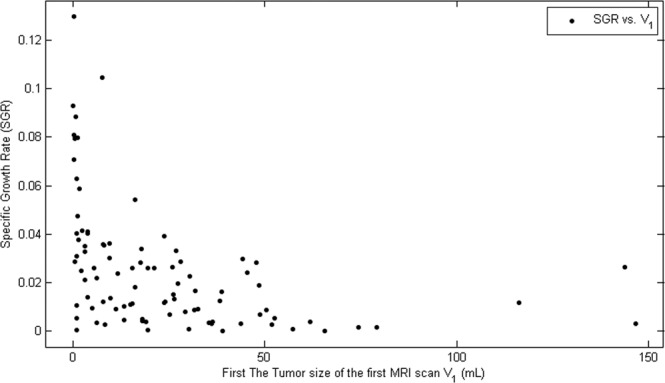
Scatter plot of SGR against tumor sizes of the first MRI scan: The horizontal axis presents the tumor size in the first MRI scan and the vertical axis presents the specific growth rate (SGR). The plot shows there is a negative correlation between the first scanned tumor volume and SGR; the variation of SGR decreases with respect to tumor size before the tumor volume reaches 100 mL.

The growth rate of individual tumors was calculated by using the specific growth rate^18^ (SGR) formula ${{\rm{SGR}}}_{{\rm{i}}}=\frac{\log \,{{\rm{V}}}_{2}^{{\rm{i}}}-\,\log \,{{\rm{V}}}_{1}^{{\rm{i}}}}{{\Delta {\rm{t}}}_{{\rm{i}}}}$, for ${\rm{i}}=1,\ldots ,94$. The scatter plot of SGR against the tumor size of first MRI scan is presented in Fig. 1.

### Model set up

From Fig. 1, there are two key observations on the growth dynamics:

1. There is a significant negative correlation between the first scanned tumor volume and SGR. Stensjøen *et al*. also pointed this out. They carried out a statistical analysis on the distribution of SGR with different tumor sizes^3^.
2. The variation of SGR decreases with respect to tumor size before the tumor size reaches 100 mL.

Our model will incorporate these two characteristics. Let ${\rm{V}}={\rm{V}}({\rm{t}})$ be the tumor size at time *t*. In most cases, phenomenological tumor growth laws are described by a single deterministic differential equation

1 $$\frac{dV}{dt}=g(V)$$

with $\,V(0)={v}_{0}$, where $g(V)$ describes the tumor growth dynamics and ${v}_{0}$ is the initial tumor volume. Typically, the model (1) has the following simplifying assumptions^9,10^:

1. The tumor has one cell population, and there is no cell heterogeneity;
2. Tumor growth is spatially independent;
3. Tumor growth does not show explicit dependence on nutrients, host vasculature, or age.

The function $g(V)$ can be expressed as the product of $V$ and the intrinsic net growth rate $\,r(V)$, thus

2 $$\frac{dV}{dt}=r(V)\,V$$

with $V(0)={v}_{0}$. It is well known that glioblastomas are composed of many cell types and are heterogeneous^19^. The heterogeneity in cell types and space may contribute to variation of intrinsic growth rate. We employ a stochastic process to express the tumor intrinsic growth rate. Let $r(V)=\alpha (V)+\sigma (V)\xi (t)$ where $\alpha (V)$ represents the deterministic intrinsic growth rate, which will be a decreasing function since growth rate decreases with tumor size, $\xi (t)$ represents white noise, and $\sigma (V)$ represents the strength of the white noise, and is a decreasing positive function. To parameterize the model, we need to undertake two steps of data fitting to determine $\alpha (V)$ and $\sigma (V)$ from our data, separately.

### Model fitting

**Figure 2:**
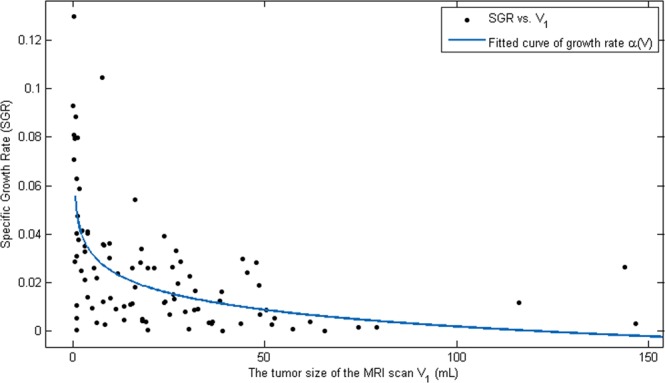
Gompertz curve fitting: The Gompertz curve, $(V)=a\,\log \,\frac{b}{V}$, is used to fit the deterministic intrinsic growth rate, where $a$ represents the intrinsic growth rate and *b* represents the carrying capacity. The best fitted values for $a=0.009916$ and $b=121.6$.

In the literature, many studies report that Gompertz curve fits for tumor growth are better than others^20–22^. Therefore, we use a Gompertz curve to fit the deterministic intrinsic growth rate, i.e., $\alpha (V)=a\,\log \,\frac{b}{V}$ where $a,b > 0$ are parameters. More concretely, *a* represents the intrinsic growth rate and *b* represents the carrying capacity. The individual conditions including nutrients, host vasculature, and age may contribute to the carrying capacity. Using pairs $({V}_{1}^{i},SG{R}_{i})$ for $\,i=1,\ldots ,94$, we obtain $a=0.009916$ and $\,b=121.6$. The fitted curve for $\alpha (V)$ is shown in Fig. 2.

**Figure 3:**
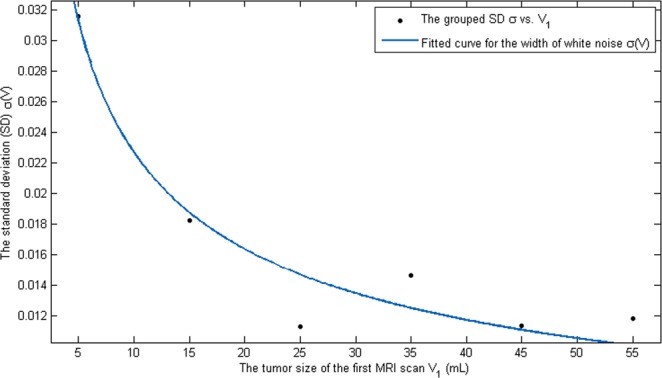
Variance fitting: White noise is used to fit the variance or standard deviation in the data set. Since there is no commonly accepted functional form for the strength of white noise, \The best fitted function is $\sigma (V)=\frac{c}{h+\sqrt{V}}$ with $c=0.0769$ and $\,h=0.2241$.

Secondly, we fit the strength of the white noise $\,\sigma (V)$. Since there is no commonly accepted functional form for that, we will find a function that is simple and fits our data well. To fit $\,\sigma (V)$, we need pairs $\,({V}_{i},{\sigma }_{i})$. For the sake of simplicity, we partition the tumor size of the first MRI scans, ${V}_{1}^{i}$, into 7 subgroups, ${D}_{j}$, for $j=1,\cdots ,7$ based on the tumor size. To be concrete, let 10, 20, 30, 40, 50, 60 be volume separators for tumor size at the first MRI scan, and ${D}_{1}=\{{V}_{1}^{k}:{V}_{1}^{k}\le 10\}$, ${D}_{2}=\{{V}_{1}^{k}:10 < {V}_{1}^{k}\le 20\},\ldots ,{D}_{7}=\{{V}_{1}^{k}:60 < {V}_{1}^{k}\le 70\}\,$. Then for each subgroup $\,{D}_{j}$, we can compute the variance ${\sigma }_{j}^{2}=\frac{1}{|{D}_{j}|-1}{\sum }_{{V}_{1}^{k}\in {D}_{j}}{({V}_{1}^{k}-{\bar{V}}_{1}^{j})}^{2}$, where ${\bar{V}}_{1}^{j}$ is the average value of $\,{D}_{j}$, and $|{D}_{j}|$ is the total number of elements in $\,{D}_{j}$. We dropped the group ${D}_{7}$ since it only has 7 data points in the range from 61.7 to 146.5. Thus, we have six ordered pairs of the form $(V,\sigma )$. Using these six ordered pairs, we find $\sigma (V)=\frac{c}{h+\sqrt{V}}$ with $c=0.0769$ and $\,h=0.2241$. The fit is shown in Fig. 3.

Therefore, we obtain the following Langevin equation^23^

3 $$\frac{dV}{dt}=aV\,\log \,\frac{b}{V}+\frac{cV}{h+\sqrt{V}}\xi (t)$$

with $V(0)={v}_{0}$, where $a=0.009916$, $b=121.6$, $c=0.0769$ and $h=0.2241$. Rewriting (3) in the usual form of a stochastic differential equation (SDE), we have

4 $$dV=\,aV\,\log \,\frac{b}{V}dt+\frac{cV}{h+\sqrt{V}}dW(t),$$

where $W(t)$ represents the standard Wiener process^24^. This is a stochastic differential equation, and is our mathematical model for glioblastoma growth.

### Model verification with the original 94 data sets from trondheim, norway

**Figure 4:**
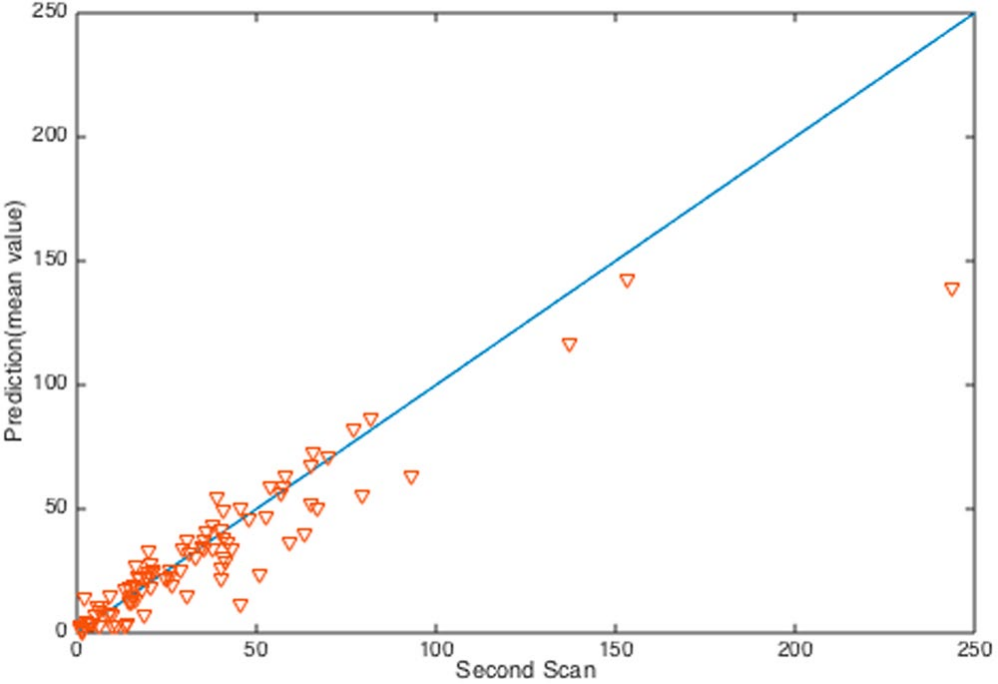
Model verification: The horizontal axis represents the second scanned tumor volume, and the vertical axis represents the tumor size predicted by our stochastic differential model. The second scanned tumor volumes were taken from the original data set from Trondheim, Norway; the first scanned tumor volumes were used to parametrize our mathematical model.

Theoretically, for a given SDE and its initial condition, we can provide a confidence region of a given probability for the tumor size at any specific future time $\,t$. For our Gompertz type diffusion model (4), since there is no closed form solution, we do not have the exact transition probability distribution for predicting tumor sizes. However, we can numerically predict the tumor size with bounds given by a prescribed probability. For example, we can predict the tumor size at future time $t$ after the first MRI scan with upper bound and lower bound given by a probability. Here we resort to numerical methods to verify our model as follows. Based on the data of the *i*-th patient, $({{\rm{V}}}_{1}^{{\rm{i}}},{\Delta {\rm{t}}}^{{\rm{i}}},{{\rm{V}}}_{2}^{{\rm{i}}})$, we use ${{\rm{V}}}_{1}^{{\rm{i}}}$ as an initial condition for the SDE (4) and then we simulate 500 solution sample paths on the time interval $(0,\Delta {t}^{i})$. From these 500 solution sample paths, we obtain an empirical distribution at time $t=\Delta {t}^{i}$ which provides the mean tumor size, ${\mu }_{i}$, and standard deviation, ${\sigma }_{i}$. We will check if $|{V}_{2}^{i}-{\mu }_{i}|\le 3{\sigma }_{i}$ for $i=1,\cdots ,94$, which means we give a 99% confidence interval. The results are shown in Fig. 4 and Table 1. Figure 4 shows our model predictions of the tumor sizes at the time the second MRI scan was done against the real tumor sizes. Table 1 shows how the real tumor sizes will fall in the region of our model prediction if we give a 99% confidence interval. As the table shows, about 60+ percent of real tumor sizes fall into our prediction intervals.

**Table 1:** Prediction of the tumor size of second MRI scan from the fitted model. Prediction of the tumor size comparing with the original data from Trondheim: Taking the initial value as the first scanned tumor size, our model predicts the tumor size after the time period from the first scan to the scan. Within 3 standard deviation or 99% confidence level, the correct prediction percentages are shown. For small tumors, the percentage of correct predictions is higher.

	Number of data	Predicted with 3 $\sigma$ error	Correct prediction percentage
Second MRI scan ${V}_{2}$	94	58	61.70%
Second MRI scan ${V}_{2} < 50$	74	50	67.57%

In addition, Table 1 summarizes the results of predictions of the tumor size of the second MRI scan to a 99% confidence level.

### Model re-verification with 3 data sets from Seattle, US

Our research group recently collected 3 untreated human glioblastoma data sets of tumor volumes at two different time points. The tumor volume was computed from three measurements in three directions that are perpendicular to each other as an ellipsoid. If we use our mathematical model with exact parameter values estimated from the original 94 data sets, two of three tumor volumes are in our model prediction range with probability 0.99 as shown in Table 2.

**Table 2:** Predictions and observations from US. Prediction of the tumor size interval comparing with the data from Seattle: Using exact parameter values estimated from data sets of Trondheim, 2 observed tumor sizes from Seattle are within the predicted interval of the tumor size with probability 0.99.

MRN	Path report	Date 1	Volume (mL)	Date 2	Volume (mL)	Prediction
U3863224	GB	8/21/2015	1.592	9/3/2015	2.715	[1.54, 3.84]
U2223925	GB	8/6/2006	20.83	8/22/2006	34.77	[22.3, 31.8]
U4348415	GB	6/19/2017	17.27	7/19/2017	29.76	[21.7, 34.9]

If we change the growth rate from the estimated value $a=0.009916$ to $\,a=0.015$, all three tumor volumes are in our prediction range with 0.99 confidence level as shown in Table 3.

**Table 3:** Observations and predictions by adjusted growth rate. Prediction of the tumor size interval comparing with the data from Seattle: Changing the intrinsic deterministic growth rate from a = 0.009916 to a =0.015, all three tumor volumes from Seattle are in our prediction range with 0.99 confidence level.

MRN	Path report	Date 1	Volume (mL)	Date 2	Volume (mL)	Prediction
U3863224	GB	8/21/2015	1.592	9/3/2015	2.715	[1.96, 4.75]
U2223925	GB	8/6/2006	20.83	8/22/2006	34.77	[25.5, 34.9]
U4348415	GB	6/19/2017	17.27	7/19/2017	29.76	[28.2, 41.6]

We do not have age records attached to the data sets. We also notice that the methods of measurements are different. The contrast-enhanced T1-weighted MRI scan was used in the data sets collected in Trondheim, while three-dimensional measurements with ellipsoid computation was used in the data sets collected in Seattle. Those methods may contribute some systematical differences in data sets^25^. In either case above, we consider our mathematical model is re-verified by these newly collected data sets.

### Ethical approval

All procedures performed in studies involving human participants were in accordance with the ethical standards of the institutional and/or national research committee and with the 1964 Helsinki declaration and its later amendments or comparable ethical standards. They were approved by the Institutional Review Board of Fred Hutchinson Cancer Research Center. The data privacy regulations were followed.

### Informed consent

Informed consent was obtained from all individual participants included in the study.

## Results

### Empirical distribution of survival times obtained by using numerical simulations

**Figure 5:**
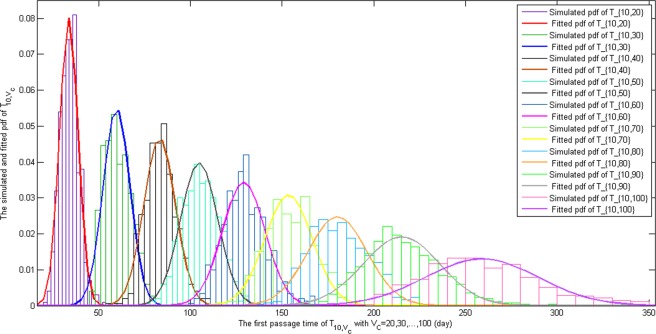
Empirical distributions of the first passage time (waiting time): ${T}_{{v}_{0},{v}_{c}}$, ${v}_{0}$ is the tumor size for which we start our observations, and ${v}_{c}$ the tumor size for which we stop our observations. Starting with ${v}_{0}=10$, ${v}_{c}=20,$ ${v}_{c}=30,$ … ${v}_{c}=100$, for each pair $({v}_{0},\,{v}_{c}),$ we simulate 500 sample paths which are plotted as histograms. The fitted probability density curves are plotted. ${T}_{{v}_{0},{v}_{c}}$ is approximately normally distributed.

Since SDE (4) can predict the tumor size at future time $t$ after the first MRI scan, we can obtain more insight on the glioblastoma growth dynamics based on this model. It is natural to explore the survival time for each patient. If we make the simplifying assumption that tumor size is the only reason for patient death, then the survival time for each patient can be expressed as the first time the tumor size reaches a specific value. We can use the first passage time random variable associated with SDE (4) to model the survival time. Since the solution of SDE (4)$\,\{V(t):t\ge 0\}$ is a diffusion process, we define the first passage time (waiting time) random variable,

$${T}_{{v}_{0},{v}_{c}}=\mathop{\inf }\limits_{t\ge 0}\{t:V(t)\ge {v}_{c}\},$$

where $V(0)={v}_{0} < {v}_{c}$, ${v}_{0}$ is the tumor size for which we start our observations, ${v}_{c}$ is the tumor size for which we stop our observations. Since there is no closed form solution to SDE (4), we study the first passage time random variable numerically. For initial tumor size $\,{v}_{0}=10$, and ${v}_{c}=20,30,\cdots ,100$, we simulate each ${T}_{{v}_{0},{v}_{c}}$ for 500 sample paths. Then, we obtain a simulated empirical distribution of $\,{T}_{{v}_{0},{v}_{c}}$. The histograms and fitted density curves are presented in Fig. 5.

From the histograms of simulated data shown in Fig. 5, the distributions of ${T}_{{v}_{0},{v}_{c}}$ are close to normal distributions. So, we assume that the distribution of the first passage time follows

$${T}_{{v}_{0},{v}_{c}} \sim Normal(\mu ({v}_{0},{v}_{c}),\sigma ({v}_{0},{v}_{c})),$$

where $\mu ({v}_{0},{v}_{c}),\sigma ({v}_{0},{v}_{c})$ are functions of ${v}_{0}\,and\,{v}_{c}$. Based on SDE (4), we consider combinations of $({v}_{0},{v}_{c})$ such that ${v}_{c}\ge {v}_{0}+10$ with ${v}_{0}=10,\,20,\,\cdots ,\,60$ and $\,{v}_{c}=20,30,\cdots ,100$. That is, we consider the tumor grows from volume ${v}_{0}$ to $\,{v}_{c}$. In total, there are 39 combinations. For each combination $\,({v}_{0},{v}_{c})$, we simulated 500 sample paths, thus we obtain 39 triples $({v}_{0},{v}_{c},\mu ({v}_{0},{v}_{c}))$ and $({v}_{0},{v}_{c},\sigma ({v}_{0},{v}_{c}))$. Table 4 shows triple of $({v}_{0},{v}_{c},\mu ({v}_{0},{v}_{c}))$ and $({v}_{0},{v}_{c},\sigma ({v}_{0},{v}_{c}))$ simulated from our model.

**Table 4:** Triples of $({v}_{0},{v}_{c},\mu ({v}_{0},{v}_{c}))$ and $({v}_{0},{v}_{c},\sigma ({v}_{0},{v}_{c}))$ from simulations. Computing the average waiting time and standard deviation of the waiting time for different initial tumor sizes and expected tumor sizes.

${{\boldsymbol{v}}}_{{\bf{0}}}$	10		20		30		40		50		60	
${{\boldsymbol{v}}}_{{\boldsymbol{c}}}$	${\boldsymbol{\mu }}$	${\boldsymbol{\sigma }}$	$\mu$	${\boldsymbol{\sigma }}$	${\boldsymbol{\mu }}$	${\boldsymbol{\sigma }}$	${\boldsymbol{\mu }}$	${\boldsymbol{\sigma }}$	${\boldsymbol{\mu }}$	${\boldsymbol{\sigma }}$	${\boldsymbol{\mu }}$	${\boldsymbol{\sigma }}$
20	33.69	4.98	—	—	—	—	—	—	—	—	—	—
30	59.80	7.25	26.20	4.95	—	—	—	—	—	—	—	—
40	83.28	8.55	50.33	6.94	24.12	4.99	—	—	—	—	—	—
50	104.77	10.04	72.64	9.19	46.88	7.11	24.03	5.56	—	—	—	—
60	129.02	11.61	95.99	11.02	69.35	10.07	47.46	8.32	23.64	6.04	—	—
70	153.48	12.96	120.95	12.44	94.82	11.98	71.54	10.95	48.13	9.12	25.77	7.46
80	180.18	16.15	149.72	16.23	124.13	15.83	99.57	14.64	76.71	14.77	54.70	12.85
90	215.12	20.88	183.13	21.97	154.76	20.17	131.79	19.80	108.89	18.16	86.83	17.49
100	258.89	30.68	224.22	29.84	196.72	25.73	173.17	27.81	153.79	26.23	127.88	26.77

Using the values in Table 4, Triples of $({v}_{0},{v}_{c},\mu ({v}_{0},{v}_{c}))$ and $({v}_{0},{v}_{c},\sigma ({v}_{0},{v}_{c}))$ from simulations. we obtain the following fitted function for $\,\mu ({v}_{0},{v}_{c}),\sigma ({v}_{0},{v}_{c})$:

5 $$\mu ({v}_{0},{v}_{c})=-\,2.643{v}_{0}+2.803{v}_{c}-10.98$$

6 $$\sigma ({v}_{0},{v}_{c})=15.28-0.1914{v}_{0}-0.3376{v}_{c}+0.001166{v}_{0}{v}_{c}+0.004797{v}_{c}^{2}.$$

**Figure 6:**
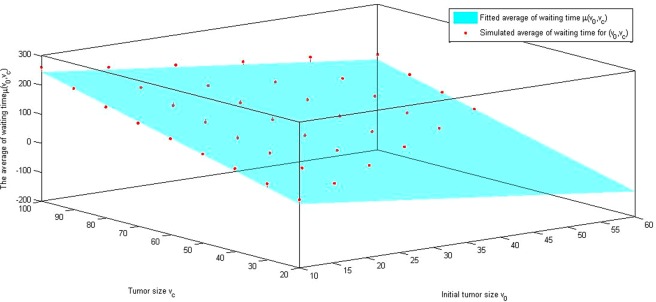
Fitted surface of the average waiting time: To find the relation between the average waiting time and tumor sizes, the initial tumor size, expected tumor size, and computed average waiting time are plotted. Estimated surface, ${\rm{\mu }}({{\rm{v}}}_{{\rm{o}}},{{\rm{v}}}_{{\rm{c}}}),$ is obtained by the best fit.

**Figure 7:**
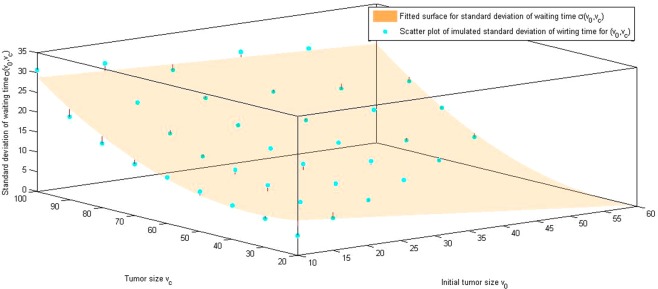
Fitted surface of standard deviation of the waiting time: To find the relation between the standard deviation of the waiting time and tumor sizes, the initial tumor size, expected tumor size, and computed standard deviation of the average waiting time are plotted. Estimated surface, ${\rm{\sigma }}({{\rm{v}}}_{{\rm{o}}},{{\rm{v}}}_{{\rm{c}}}),$ is obtained by the best fit.

Figure 6 shows the fitted surface $\mu ({v}_{o},{v}_{c})$ for the average waiting time, which depends on initial tumor size, ${v}_{o}$, and stopping tumor size, ${v}_{c}$, based on the simulated data in Table 4. Figure 7 shows the fitted surface $\sigma ({v}_{0},{v}_{c})$ for the standard deviation of the waiting time, which depends on initial tumor size, ${v}_{o}$, and tumor stopping size (stopping observation), ${v}_{c}$, based on the simulated data in Table 4.

### Application of our model: theoretical surgery strategies

The extent of resection has been an important subject in neurosurgery research and practice. It has only recently been established that there is a statistical correlation between the extent of resection and survival^11–15,26^. However, almost all of the studies are retrospective and thus subject to numerous sources of bias and variation. Furthermore, there are only three categories for the extent of resection: stereotactic biopsy, subtotal resection, and gross total resection (radical or complete resection). In clinics, tumors in some locations may not be amenable to gross total resection due to proximity to functional tissue. For example, the relation of the tumor to eloquent cortex (i.e. primary motor cortex or speech centers) is important since it directly corresponds to the proportion of the tumor that can be resected without permanent morbidity^11^. For elderly patients, the optimal extent of resection remains uncertain because of potential higher rates of mortality and morbidity associated with more extensive degrees of resection^12^. The choice of optimal extent of resection may depend on the tumor size and location, the patient’s general and neurological status, and the neurosurgeon’s experience^13^. Although there are some prospective studies^15,27,28^, a prospective study with quantifying extent of resection is necessary.

Our SDE model is a good tool to study extent of resection prospectively. We can explore the survival time after surgical resection and may help surgeons to choose a best protocol for the treatment based on an individual patient’s conditions, such as tumor size and location and possible resection proportion (quantified extent of resection). In the following, we will explore how various combinations of different diagnosed tumor sizes, tumor sizes when surgery is performed, different resection proportion, and repeated resections, will affect the patient survival time.

We assume that the glioblastoma growth pattern after resection is the same as before the resection. Specifically, the waiting time ${T}_{1}={T}_{{v}_{0},{v}_{c}}$ of tumor growing from the size $\,{v}_{0}$ to size $\,{v}_{c}$ and the waiting time ${T}_{2}={T}_{{v}_{c}\text{'},{v}_{T}}$ of tumor growing from the size $\,{v}_{c\text{'}}$ after resection to the death size $\,{v}_{T}$ follow the normal distribution with the mean $\,\mu ({v}_{0},{v}_{c})$ and $\,\mu ({v}_{c\text{'}},{v}_{T})$, and the standard deviation $\,\sigma ({v}_{0},{v}_{c})$ and $\,\sigma ({v}_{c\text{'}},{v}_{T})$, respectively. That is, ${T}_{1} \sim N(\mu ({v}_{0},{v}_{c}),\sigma ({v}_{0},{v}_{c}))$ and $\,{T}_{2} \sim N(\mu ({v}_{c\text{'}},{v}_{T}),\sigma ({v}_{c\text{'}},{v}_{T}))$.

Now, we can address the following four questions

1. How long could a patient survive without treatment when the initial diagnosed tumor size is $\,{v}_{0}$?
2. Long could a patient survive if the patient accepts surgical resection immediately when the initial diagnosed tumor size is ${v}_{0}$ with the resection proportion *c*%?
3. How long could a patient survive if the patient accepts surgical resection when the tumor reaches a size ${v}_{c}\,$for which $\,{v}_{0} < {v}_{c} < {v}_{T}$?
4. Under some conditions, if a second surgical resection is needed, how long could a patient survive?

### The first question

Let the random variable ${T}_{1}={T}_{{v}_{0},{v}_{T}}$ be the survival time when the tumor grows from the initial diagnosed size ${v}_{0}$ to the death size $\,{v}_{T}.\,$It is assumed to follow the normal distribution $\,N(\mu ({v}_{0},{v}_{T}),\sigma ({v}_{0},{v}_{T}))$. In Table 5, we present the expectation and standard deviation (SD) of the survival time for a patient without treatment with an initial diagnosed tumor size $\,{v}_{0}$, where we take the initial diagnosed tumor size from 1 mL to 50 mL for demonstration, and the unit of the survival time is days. It is easy and obvious to conclude that small glioblastomas have longer survival times.

**Table 5:** Expected survival time (SD) without treatment (days). Expected survival time and standard deviation: 100 mL is taken to be a death size of the tumor in order to compute the expectation of the survival time. For example, if a tumor of 1 mL is observed, our model predicts the average survival time is 267 days, and its standard deviation is 29 days. Notice that the estimated death size is 121.6 mL.

${{\boldsymbol{v}}}_{{\bf{0}}}$	1	2	5	10	20	30	50
Expectation (Standard Deviation)	267(29)	264(29)	256(29)	242(29)	216(28)	190(27)	137(26)

### The second question

For the second question, we use the random variable ${T}_{2}={T}_{v{\prime} ,{v}_{T}} \sim N(\mu (v{\prime} ,{v}_{T}),\sigma (v{\prime} ,{v}_{T}))$, where $v{\prime} ={v}_{0}(100-c) \%$ and $c \%$ is cut-off percentage. In Table 6, we present our computation results for the expectation and standard deviation of the survival time $\,{T}_{2}={T}_{v\text{'},{v}_{T}}$ for a patient who accepts the surgical resection immediately after tumor diagnosis and the resection proportion is $\,c \%$. We only present the cases where the diagnosed tumor size has volume from 1 mL to 50 mL and the resection proportions are percentages from 50 to 98. From our computation, we learn that a surgical resection does not increase survival times much for small tumors no matter what percentage of the tumor can be cut off, while for large tumors the more a surgical resection can cut off from the tumor, the longer will be the survival time for the patient. In the clinical study^14^, the authors reported that a high extent of resection improves overall survival, and even for 78% of resection extent, it also gives survival benefits, which agrees with our conclusions.

**Table 6:** Expected survival time (SD) with immediate resection (days). Expected survival time under immediate resection: Our computation provides average survival times under different resection percentage. For example, for a tumor of 50 mL, if no resection is performed, the patient has an average survival of 137 days with standard deviation 26 days; if the resection with 50 percent is performed, the average survival is 203 days with standard deviation 28 days; if the resection with 90 percent is performed, the average survival is 256 days with standard deviation 29 days.

	1	2	5	10	20	30	50
0%	267(29)	264(29)	256(29)	242(29)	216(28)	190(27)	137(26)
50%	268(29)	267(29)	263(29)	256(29)	243(29)	230(28)	203(28)
60%	268(29)	267(29)	264(29)	259(29)	248(29)	238(29)	216(28)
70%	269(29)	268(29)	265(29)	261(29)	253(29)	246(29)	230(28)
80%	269(29)	268(29)	267(29)	264(29)	259(29)	253(29)	243(29)
90%	269(29)	269(29)	268(29)	267(29)	264(29)	261(29)	256(29)
98%	269(29)	269(29)	269(29)	269(29)	268(29)	268(29)	267(29)

### The third question

For the third question, the tumor grows for some period of time from the initial diagnosed size $\,{v}_{0}$ to size $\,{v}_{c}\,$and then a surgical resection is performed. The survival time is ${T}_{1}+{T}_{2}$ where ${T}_{1} \sim N(\mu ({v}_{0},{v}_{c}),\sigma ({v}_{0},{v}_{c}))$ and ${T}_{2} \sim N(\mu ({v}_{c{\prime} },{v}_{T}),\sigma ({v{\prime} }_{c},{v}_{T}))$ with ${v{\prime} }_{c}={v}_{c}(100-c) \%$. Since the survival time is a sum of two random variables, we need to consider how these two random variables affect each other and their impact on the survival time. To include the relation between these two random variables, we use the correlation coefficient ρ. From the property that the sum of normally distributed random variables is still a normally distributed random variable, we know that

$${T}_{1}+{T}_{2} \sim N(\mu {\prime} ,\sigma {\prime} ),$$

where, $$\sigma {\prime} =\sqrt{{\sigma }^{2}({v}_{0},{v}_{c})+{\sigma }^{2}({v{\prime} }_{c},{v}_{T})+2\rho \sigma ({v}_{0},{v}_{c})\sigma ({v{\prime} }_{c},{v}_{T})}$$ and $$\mu {\prime} =\mu ({v}_{0},{v}_{c})+\mu ({v{\prime} }_{c},{v}_{T})$$.

In this computation, there are four parameters: the initial diagnosed tumor size $\,{v}_{0}$, the tumor size $\,{v}_{c}$ when surgical resection is performed (after random time $\,{T}_{1}$), the resection proportion $\,c \%$, and the correlation coefficient ρ. To demonstrate, we choose the initial diagnosed tumor size to be $\,{v}_{0}=1\,{\rm{mL}}$, then let the tumor grow to size ${v}_{c}$ for surgical resection with cut-off percentage c%. To compute the survival time, we choose three values for the correlation coefficient ρ for each theoretical treatment. Table 7 shows the expectation and standard deviation of the survival time for the parameter values we choose.

**Table 7:** Expected survival time (SD) after tumor growth then resection (days). Expected survival time after tumor growth then resection: Assume tumor initial size is 1 mL. If resection is immediately performed, the average survival is 268 days with SD 29 days. Letting it grow to 50 mL, then resection is performed with different percentages, the average survival is from 330 days to 393 days; the standard deviations vary according to the correlation coefficient ρ between the growth time T_1_ and survival time T_2_.

ρ		2	5	10	20	30	50
0	50%	259(33)	263(32)	271(32)	285(31)	300(30)	330(29)
0.5	50%	259(39)	263(38)	271(37)	285(35)	300(34)	330(34)
0.8	50%	259(42)	263(41)	271(40)	285(37)	300(36)	330(35)
0	60%	259(33)	264(32)	273(32)	291(31)	308(30)	343(30)
0.5	60%	259(39)	264(38)	273(37)	291(35)	308(34)	343(34)
0.8	60%	259(42)	264(41)	273(40)	291(38)	308(36)	343(35)
0	70%	260(33)	266(32)	276(32)	296(31)	316(30)	356(30)
0.5	70%	260(39)	266(38)	273(37)	296(35)	316(34)	356(35)
0.8	70%	260(42)	266(41)	273(40)	296(38)	316(37)	356(36)
0	80%	260(33)	267(32)	278(32)	301(31)	328(30)	369(31)
0.5	80%	260(39)	267(38)	278(37)	301(35)	328(35)	369(35)
0.8	80%	260(42)	267(41)	278(40)	301(38)	328(37)	369(36)
0	90%	261(33)	268(32)	281(32)	306(31)	332(30)	383(31)
0.5	90%	261(39)	268(38)	281(37)	306(35)	332(35)	383(35)
0.8	90%	261(42)	268(41)	281(40)	306(38)	332(37)	383(37)
0	98%	261(33)	269(32)	283(32)	311(31)	338(31)	393(31)
0.5	98%	261(39)	269(38)	283(37)	311(35)	338(35)	393(36)
0.8	98%	261(42)	269(41)	283(40)	311(38)	338(37)	393(37)

From our computation, we can conclude that for a small tumor, if we wait until the tumor grew to a big tumor and then perform surgical resection, the survival time would increase. If two survival times are positive linear related, the variance of the survival time will increase; if they are negative linear related, then the variance of the survival time will decrease. It may be reasonable to assume the survival times are positive linear related. However, the mean survival time keeps the same.

### The fourth question

We consider a second surgical resection. Based on the procedure in the third question, after the first surgical resection for some period of time the tumor will grow to size $\,{v}_{{c}_{2}}$, then a second surgical resection is performed. After the second resection, if the random time for the tumor growth to death size is $\,{T}_{3}$, the survival time for the patient is as follows

$${T}_{1}+{T}_{2}+{T}_{3}.$$

This is a normally distributed random variable with distribution $\,N(\mu {\prime\prime} ,\sigma \text{'}\text{'})$, where $\mu {\prime\prime} =\mu ({v}_{0},{v}_{{c}_{1}})+\mu ({v}_{{c{\prime} }_{1}},{v}_{{c}_{2}})+\mu ({v}_{{c{\prime} }_{2}},{v}_{T})$ and $\sigma {\prime\prime} ={{\boldsymbol{\sigma }}}^{{\boldsymbol{T}}}{\boldsymbol{R}}{\boldsymbol{\sigma }}$ with $$\sigma =(\begin{array}{c}\sigma ({v}_{0},{v}_{{c}_{1}})\\ \sigma ({v}_{{c{\prime} }_{1}},{v}_{{c}_{2}})\\ \sigma ({v}_{{c{\prime} }_{2}},{v}_{T})\end{array})$$ and $$\,R=(\begin{array}{ccc}1 & {\rho }_{12} & {\rho }_{13}\\ {\rho }_{12} & 1 & {\rho }_{23}\\ {\rho }_{13} & {\rho }_{23} & 1\end{array})$$.

In this computation, there are eight parameters: the initial diagnosed tumor size $\,{v}_{0}$, the tumor size $\,{v}_{{c}_{1}}$ when the first surgical resection is performed (after random time $\,{T}_{1}$), the first resection proportion $\,{c}_{1} \%$, the tumor size ${v}_{{c}_{2}}\,$when the second surgical resection is performed (after random time $\,{T}_{2}$), the second resection proportion $\,{c}_{2} \%$, and three correlation coefficients ${\rho }_{12},{\rho }_{13},{\rho }_{23}$ within $\,{T}_{1}$, $\,{T}_{2}$ and $\,{T}_{3}$. To demonstrate and simplify presentation, we choose the initial diagnosed tumor size $\,{v}_{0}$=1 mL, the first and second resection proportion $\,{c}_{1} \% =80 \%$ and $\,{c}_{2} \% =50 \%$, and give the triple $({\rho }_{12},{\rho }_{13},{\rho }_{23})$ three choices. For the tumor sizes ${v}_{{c}_{1}}\,$and ${v}_{{c}_{2}}$, we choose some volume from 2 mL to 50 mL and 15 ml to 90 mL respectively. Table 8 shows the expectation and standard deviation of the survival time for these parameter values.

**Table 8:** Expected survival time (SD) for two resections (days). Expected survival time after two resections: Assume tumor initial size is v_0_. After random time T_1_, the tumor reaches size v_c1_, then resection with c_1_%; then after another random time T_2_, the tumor reaches size v_c2_, the second resection with c_2_%; then the patient survives with some period of time T_3_. To demonstration, taking v_0_ = 1 mL, c_1_% = 80%, c_2_% = 50%, and correlation coefficients ρ_12_, ρ_13_, ρ_23_ among T_1_, T_2_ and T_3_. For example, the total survival time will be 492 days if the first resection with 80% is performed when the tumor reaches 50 mL and the second resection with 50% is performed when the tumor reaches 90 mL.

${\boldsymbol{(}}{{\boldsymbol{\rho }}}_{{\bf{12}}}{\boldsymbol{,}}{{\boldsymbol{\rho }}}_{{\bf{13}}}{\boldsymbol{,}}{{\boldsymbol{\rho }}}_{{\bf{23}}}{\boldsymbol{)}}$		$2$	5	10	20	40	50
$(0,0,0)$	15	271(34)	278(34)	290(33)	312(32)	358(32)	381(32)
$(0.5,0,0.5)$	15	271(41)	278(40)	290(39)	312(38)	358(37)	381(38)
$(0.8,0,0.8)$	15	271(44)	278(44)	290(43)	312(41)	358(40)	381(40)
$(0,0,0)$	20	279(34)	286(33)	297(33)	320(32)	365(32)	388(32)
$(0.5,0,0.5)$	20	279(41)	286(40)	297(39)	320(38)	365(37)	388(38)
$(0.8,0,0.8)$	20	279(43)	286(43)	297(42)	320(40)	365(39)	388(39)
$(0,0,0)$	40	309(33)	315(32)	327(32)	349(31)	395(31)	418(31)
$(0.5,0,0.5)$	40	309(38)	315(38)	327(37)	349(36)	395(35)	418(35)
$(0.8,0,0.8)$	40	309(41)	315(41)	327(40)	349(39)	395(38)	418(38)
$(0,0,0)$	50	323(33)	330(32)	342(32)	364(31)	410(31)	433(31)
$(0.5,0,0.5)$	50	323(39)	330(38)	342(38)	364(37)	410(36)	433(36)
$(0.8,0,0.8)$	50	323(42)	330(42)	342(41)	364(40)	410(39)	433(39)
$(0,0,0)$	70	353(34)	360(34)	371(33)	394(32)	439(32)	462(32)
$(0.5,0,0.5)$	70	353(42)	360(42)	371(41)	394(40)	439(39)	462(39)
$(0.8,0,0.8)$	70	353(46)	360(46)	371(45)	394(44)	439(43)	462(43)
$(0,0,0)$	80	368(36)	375(35)	386(35)	409(34)	454(33)	477(34)
$(0.5,0,0.5)$	80	368(45)	375(45)	386(44)	409(43)	454(42)	477(42)
$(0.8,0,0.8)$	80	368(50)	375(49)	386(49)	409(47)	454(46)	477(47)
$(0,0,0)$	90	383(38)	389(38)	401(37)	424(37)	469(36)	492(36)
$(0.5,0,0.5)$	90	383(49)	389(49)	401(48)	424(47)	469(46)	492(46)
$(0.8,0,0.8)$	90	383(55)	389(54)	401(53)	424(52)	469(51)	492(51)

From our computation, we may conclude that for a small tumor, if we wait until it grows to a large tumor and then perform a surgical resection with a high cut-off percentage, and wait again until the tumor grows to an even larger tumor then perform a second surgical resection, the patient survival time will significantly increase. We also conclude that correlation coefficients within waiting times for tumor growth do not have effect much on the survival time. This confirms the results reported in the studies^16,17^.

Our computational investigation first confirms the intuitively expected result that small glioblastomas have a longer survival time while large glioblastomas have a short survival time. We also confirm that the patient will have survival benefits even if the extent of resection is 70%. Our computation thirdly confirms that a second resection will increase patient survival time. In addition, we obtain the following conclusions. For large glioblastomas, the more a surgical resection can cut off from the tumor, the longer will be the patient survival time. For small glioblastomas, if we wait until the glioblastoma grows to a large tumor and then perform surgical resection, the patient survival time will increase; furthermore, if we wait again until the glioblastoma grows to an even larger tumor and then perform a second surgical resection, the patient survival time will increase further.

It is clear that our theoretical study provides ample sources of thought for the surgical management of glioblastomas, and while our predictions provide results that are intuitively obvious, they quantify the possible benefits of resections. There are many factors in clinical practice which should be taken into consideration when we make decisions. For example, waiting for a glioblastoma in the brain to get bigger is not only dangerous in terms of letting cells penetrate deep into the tissue but also dangerous in terms of acute symptoms due to increased brain pressure. This requires us to consider parameters such as metastasis, extent of spatial heterogeneity, and invasiveness. Most importantly, the genetics of the tumors should be considered^29,30^.

## Discussion

The clinical data on the growth rate of untreated glioblastomas show a large variability among different patients^3^. Several deterministic mathematical models have been proposed to describe glioma growth patterns. However, these mathematical models are unable to describe and predict the variance among patients (or experimental animals). Based on a data set of 94 glioblastoma patients, we find the best fitted mathematical model based on Gompertz growth with white noise. We use white noise to model the variability among patients and random factors in tumor cell environment. The strength coefficient of the white noise we fitted is of fraction form. Instead of growth curves predicted by deterministic models, we predict a band where the center curve is the mean of the solution (the tumor size), and its width is given by a prescribed probability that describes the variance of the tumor size deviating from the average over time. As mentioned in the Materials and Methods section, almost 2/3 of glioblastoma growth in the data set from Trondheim, Norway, falls into our predicted band. Newly collected human glioblastoma data from Seattle, US, also fall into our model prediction band after the growth rate is increased. Although our stochastic differential equation model captures some variability of the growth dynamics of glioblastomas, we cannot expect a model as simple as the one we propose to hold for all human glioblastomas, which have great heterogeneity among individual patients.

Since there is no closed form solution of our stochastic differential equation model, we numerically simulate our model for a large number of solution paths, and obtain some empirical results. One result is the empirical distribution of the survival time. We find it to be a normally distributed random variable, with mean and variance dependent on the tumor sizes. From our computational simulations, we numerically fit simple formulas to calculate the mean and variance in terms of tumor sizes. If the tumor size of a glioblastoma patient is found from an MRI scan, our empirical distribution of the survival time can predict how long this patient can live without treatment under a given probability, and can also calculate their average survival time and variance. This may give physicians and patients some information to prepare for a better life in recovery.

One interesting question is: what is the best time for surgical resection when a patient is diagnosed with a glioblastoma? This problem involves several parameters including tumor size, tumor location in the brain, physician’s experience, possible extent of surgical resection estimated, etc. For example, for a patient with a glioblastoma, each physician has their own judgment of how much they can cut the tumor based on their experience. As a theoretical study, we consider the percentage of the tumor to be removed which is quantified extent of resection, the timing of surgical resections, and times of surgical resections. We confirm an obvious conclusion that small glioblastomas have a longer survival time while large glioblastomas have a short survival time. We also confirm that the patient will have survival benefits even if the extent of resection is low. Our computation shows that for large glioblastomas, the higher the percentage a surgical resection can cut off from the tumor, the longer will be the patient survival time. However, for small glioblastomas, a surgical resection does not increase survival time much no matter what percentage of the tumor can be cut off. If we wait until a small glioblastoma grows to a large tumor and then perform surgical resection, the patient can attain a longer survival time; furthermore, if we wait again until the glioblastoma grows to an even larger tumor and then perform a second surgical resection, the patient will get a significant longer survival time. We may conclude that repeated surgical resections will give more benefits to patients theoretically. In fact, the reoperation has been done in some clinics and experiments^16,17^. A recent systematic review and meta-analysis of contemporary literature suggests that repeated surgery at glioblastoma recurrence in select patients confers a significant, prognostic overall survival advantage independent of other prognostic factors^31^. These newer studies are significantly more likely to suggest greater benefit than are older studies, while larger prospective randomized controlled studies are needed to validate these findings^31^.

It is clear that our results are based on our simple mathematical model and its assumptions. These assumptions do not take all necessary factors into account and so our model predictions must be treated with great caution. For instance, we assume the tumor size determines patient survival. However, in many cases, the real driver of survival is the genetics of the tumors^29,30^ which may outweigh the tumor size at diagnosis. In the theoretical resection, we also assume that resection does not alter growth dynamics. This may not be true in some cases since the neurological conditions may change after resection. The result that the patient can attain a longer survival time when a small glioblastoma grows to a large tumor and then a surgical resection is performed, may be explained by observing that the time for a small tumor to grow to a large tumor added to the time that the patient survives after the resection is longer than the time taken for a small tumor to grow to a small or medium tumor plus the time that the patient survives after the resection. However, glioblastomas are known to be spatially diffuse, so that by allowing a tumor to grow, we risk potentially fatal spread to other parts of the brain. It is clear that, in neurosurgery, the aim of resection is always total resection, but without impacting on patient’s neurological function, and to minimize the risk of new postoperative deficits such as brain mapping/stimulation, navigation with diffusion tensor imaging. The next step in the type of analysis presented here would be to link the tumor growth rate with the genetic information and to develop a spatially extended model or adapt some of the models in the literature^32–35^ so that one could apply some of the ideas from the present paper. However, that would involve a significant extension and is beyond the scope of the present paper.

In addition, we did not consider other treatments which can be easily incorporated into our model. Surgery is usually combined with other treatments, such as radiotherapy and chemotherapy. When relevant clinical or experimental data are available, we should be able to incorporate other treatments into our stochastic differential equation model or into a spatially extended stochastic differential equation model, and provide more insight into glioblastoma growth and treatments.

## Conclusion

Human untreated glioblastoma growth *in vivo* may follow a new type of Gompertz diffusion dynamics, which is described by a stochastic differential equation. Our mathematical model not only captures the average growth pattern and the variance among individual patients, but also can predict, in many cases, individual glioblastoma growth paths. The empirical distribution of survival times simulated from our mathematical model can be used to calculate a patient’s survival time with a prescribed probability. We obtain empirical formulas to easily calculate the average survival time and its variance. As proof of principle, our mathematical model can be used to provide different protocols for performing surgical resections according to tumor sizes which will give patients long survival times. The conclusion should be interpreted with caution, owing to the number of simplifications of the mathematical modeling and small size of the data set.
